# Vitamin D and SARS-CoV-2 infection—evolution of evidence supporting clinical practice and policy development: A position statement from the Covit-D Consortium

**DOI:** 10.1007/s11845-020-02427-9

**Published:** 2020-11-21

**Authors:** Daniel M. McCartney, Paula M. O’Shea, John L. Faul, Martin J. Healy, Greg Byrne, Tomás P. Griffin, James Bernard Walsh, Declan G. Byrne, Rose Anne Kenny

**Affiliations:** 1 School of Biological and Health Sciences, College of Sciences & Health, Technological University Dublin - City Campus, Dublin 8, Ireland; 2 Department of Clinical Biochemistry, Galway University Hospitals, Galway, Ireland; 3 School of Medicine, National University of Ireland Galway, Galway, Ireland; 4 James Connolly Memorial Asthma Research Centre, Royal College of Surgeons in Ireland, Connolly Hospital Blanchardstown, Dublin 15, Ireland; 5 Biochemistry Department, St. James’s Hospital, Dublin 8, Ireland; 6 Regenerative Medicine Institute at CÚRAM SFI Research Centre, School of Medicine, National University of Ireland Galway, Galway, Ireland; 7 Centre for Endocrinology, Diabetes and Metabolism, Galway University Hospitals, Galway, Ireland; 8 Mercer’s Institute for Successful Ageing, St James’s Hospital, Dublin 8, Ireland; 9 Department of Medical Gerontology, School of Medicine, Trinity College Dublin, Dublin 2, Ireland; 10 Medicine Directorate, St. James’s Hospital, Dublin 8, Ireland

## Introduction

**Fig. 1:**
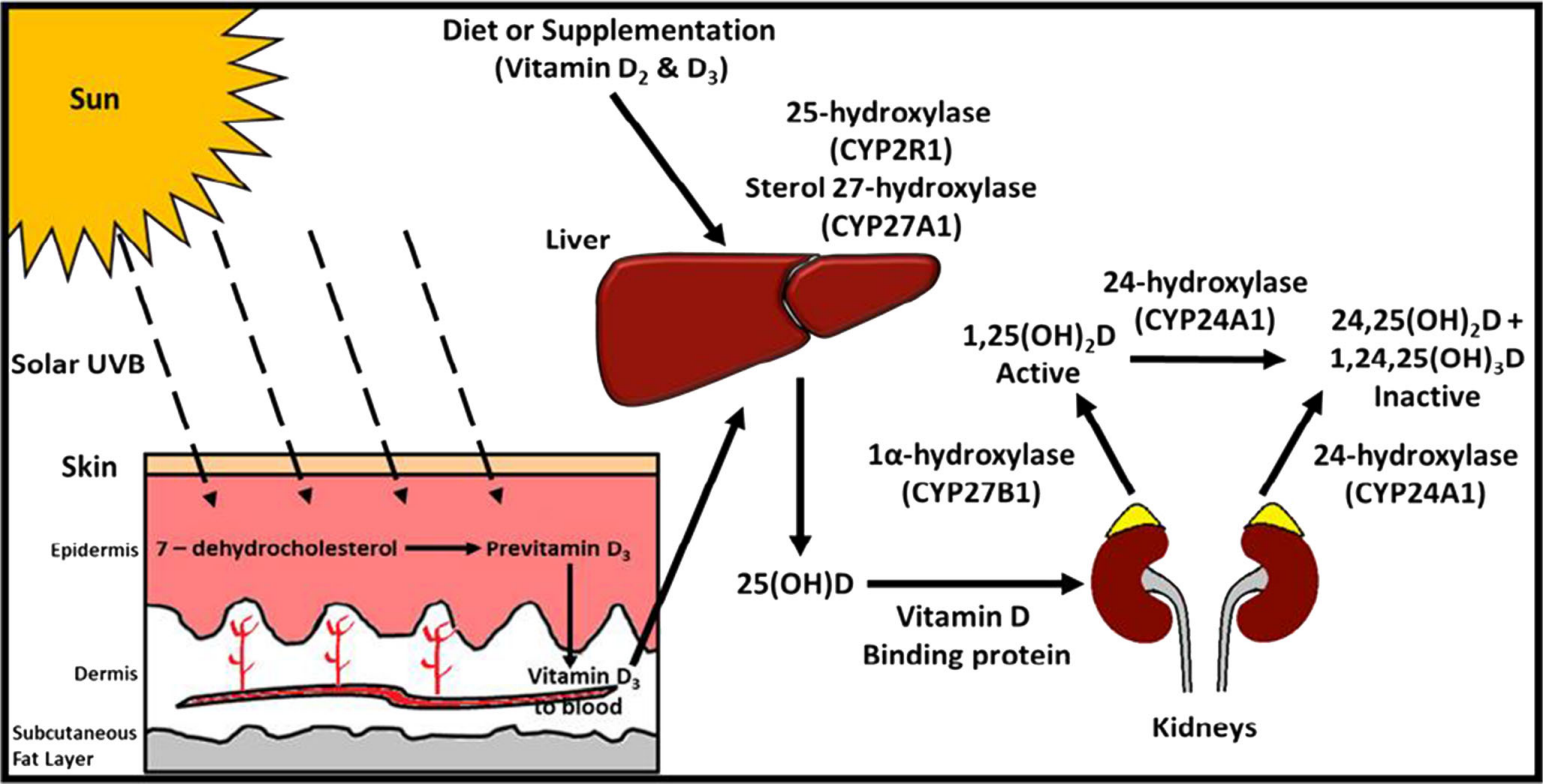
Vitamin D metabolism: UVB radiation penetrates the skin, converting 7-dehydrocholesterol to pre-vitamin D_3_, which is rapidly converted to vitamin D_3_. Vitamin D_3_ is transported through the circulation to the liver. Dietary vitamin D_2_ and D_3_ are transported from the intestine to the liver by chylomicrons (plasma and lymph). In the liver, vitamin D is hydroxylated to 25(OH)D, mediated by CYP2R1 (cytochrome P450 [CYP] enzyme). Once released into the circulation, 25(OH)D binds to vitamin D binding protein and is transported to the kidneys and other tissues. In the proximal tubules of the kidney, 1α-hydroxylation (CYP27B1) of 25(OH)D results in the production of the active vitamin calcitriol (1,25(OH)_2_D). 1,25(OH)_2_D induces the expression of the enzyme 24-hydroxylase encoded by the CYP24A1 gene which catalyses the conversion of 25(OH)D and 1,25(OH)_2_D to the inactive 24-hydroxylated products, 24,25(OH)_2_D and 1,24,25(OH)_3_D respectively. Adapted with permission from Griffin et al. [1]

Vitamin D is a vitamin pro-hormone which can be taken exogenously from the diet or supplements, or can be synthesised cutaneously through the action of summer sunlight on skin as shown in Fig. 1 [1]. While oral intake is an important source of vitamin D, the major physiological source is UVB irradiation at a wavelength of 290–315 nm [2]. The ability of the skin to synthesise vitamin D is compromised at northerly latitudes, particularly amongst those who are older or who have dark skin pigmentation.

While the kidneys are the major site of 1α-hydroxylation of 25(OH)D to its bio-active metabolite 1,25(OH)_2_D, it is now known that activation of vitamin D also occurs in many other cells and tissues. These include cellular components of both the innate and adaptive immune systems, highlighting the role of vitamin D as an important immunomodulator [3].

Susceptibility to viral infection and disease severity during viral infection are largely determined by host characteristics which influence immune response. For example, demographic characteristics, such as age and ethnicity, appear to greatly influence survival when patients are infected by the newly described SARS-CoV-2 agent. Children less than 10 years of age have an extraordinarily low mortality rate of 0.01%, while the very old (greater than 80 years) have greater disease severity and a mortality rate of close to 12.5% [4]. Patients with obesity have a 50% increased mortality rate [5], while black patients have approximately twice the mortality of white patients [6]. These differences are not entirely explained by the presence of comorbid illnesses such as diabetes mellitus and hypertension, conditions which are also commoner in those with obesity, and in older and black populations. We and others have hypothesized that nutritional state, and in particular vitamin D deficiency, which is also highly prevalent in those with obesity, and in older and black populations, might also affect infective risk and disease severity through its effects on immune function.

In the absence of a cure, or an effective safe vaccine for SARS-CoV-2, it is timely to consider whether vitamin D deficiency is an easily reversible host factor which increases the risk of SARS-CoV-2 infection and worsens disease severity, and by extension, whether optimisation of vitamin D status through supplementation can ameliorate these risks.

In the context of the accumulating evidence outlined in this position statement which strongly suggests a protective role for vitamin D against SARS-CoV-2 infection and Covid-19 disease severity, this paper is a call to action for Health Professionals and Policy Makers in Ireland to:

(i) Recognise the importance of enhanced vitamin D status in skeletal and extra-skeletal health, and particularly in the optimisation of immune response;
(ii) Identify more adults with vitamin D deficiency through more widespread measurement of serum 25(OH)D;
(iii) Prescribe vitamin D at doses which achieve a restoration of 25(OH)D levels to greater than 50 nmol/l, with a confirmatory subsequent blood draw to ensure restoration has been achieved;
(iv) Develop explicit population guidance and clinical protocols for vitamin D supplementation at these effective doses, as part of a comprehensive policy response to combat vitamin D deficiency and enhance the immune function and overall health of the Irish population.

## Suggestive background evidence

Herein, we describe several emerging lines of evidence from disparate scientific fields which have examined the links between vitamin D status and the likelihood of SARS-CoV-2 infection and Covid-19 disease severity. While some of these studies are associative only and do not necessarily infer a causal relationship, collectively they support the over-arching premise that vitamin D deficiency increases the risks associated with SARS-CoV-2 infection, and that optimisation of vitamin D status is protective against these risks.

The evidence to support this position is presented hierarchically as follows:

- circumstantial evidence from studies of Covid-19 patient demographics;
- evidence of an overlap in the inflammatory processes which characterise vitamin D deficiency and severe Covid-19 disease;
- evidence from prior observational and interventional studies investigating respiratory infection outcomes in patients according to vitamin D status and vitamin D supplementation;
- evidence from national population studies comparing the prevalence of SARS-CoV-2 infection and Covid-19 mortality with whole country serum 25(OH)D levels,
- evidence from studies comparing the rate of SARS-CoV-2 detection according to vitamin D status,
- evidence from case-control studies examining clinical outcome in Covid-19 patients according to vitamin D status, and
- evidence from intervention studies showing reduced disease severity and reduced mortality in Covid-19 patients receiving vitamin D supplementation.

### Circumstantial evidence from studies of Covid-19 patient demographics

Since the publication of the first literature linking low serum vitamin D levels with increased Covid-19 disease severity at the beginning of April 2020 [7–9], some 270 further studies on the topic have appeared in the peer-reviewed literature. The initial studies associating vitamin D status and Covid-19 severity were observational and focussed on the preponderance of Covid-19-related morbidity and death in patients with characteristics also associated with vitamin D deficiency (e.g. obesity, darker skin pigmentation, pre-existing medical conditions, older age) [9, 10].

### Evidence of an overlap in the inflammatory processes that characterise vitamin D deficiency and severe Covid-19 disease

The possibility that vitamin D deficiency causally increases disease severity amongst some patients infected with SARS-CoV-2 is biologically plausible, given the distinct immunophenotype and other biochemical aberrations which characterise both severe Covid-19 disease and vitamin D deficiency. These include pronounced interleukin-6 (IL-6) and tumour necrosis factor-alpha (TNF-α) elevation, exaggerated interferon-gamma (IFNγ) response and a shift towards amplified Th1 adaptive immune responses, as well as angiotensin-converting enzyme 2 (ACE2) suppression and increased coagulability [11–18]. These shared characteristics support the idea that low levels of vitamin D might accentuate at least some parts of the “cytokine storm” and other associated biochemical abnormalities which are typically seen in patients with severe Covid-19 disease.

### Evidence from prior observational and interventional studies investigating respiratory infection outcomes in patients according to vitamin D status and vitamin D supplementation

Multiple observational and interventional studies, meta-analyses, and systematic reviews have attempted to establish whether a higher risk of all acute respiratory infections (ARI), including viral infection, occurs in those with lower vitamin D levels [19], and whether a lower risk for all ARIs occurs in those who are supplemented with vitamin D [20–22]. While some studies show that vitamin D status has an effect, others do not. Notwithstanding these inconsistencies, however, it is noteworthy that the proposed protective effects of supplementation do appear to be more pronounced amongst those with low vitamin D levels at baseline [20, 22].

There are also prospective observational data which suggest a role for vitamin D deficiency in increased respiratory mortality, including that mediated by fulminant respiratory infection. Longitudinal data in 10,000 German adults aged 50–75 years followed for 15 years showed 2.1 times higher respiratory mortality amongst those with 25(OH)D of 30–50 nmol/l and 3.0 times higher respiratory mortality in those with 25(OH)D < 30 nmol/l when compared to patients with a 25(OH)D greater than 50 nmol/l. Statistically, after adjustment for sex, age, season of blood draw, school education, smoking, BMI, physical activity, and fish consumption, 41% of the variability in respiratory mortality during this 15-year follow-up period was independently associated with 25(OH)D levels < 50 nmol/l [23].

Overall, while the data appear to favour a protective role for vitamin D supplementation against respiratory infection, especially in those with low vitamin D levels at baseline, and especially with daily and weekly dosing [20], the absence of unanimity in this regard may reflect not just methodological differences between these studies, but also the wide spectrum of infections that affect the respiratory system from year to year, season to season, and between different populations.

### National population studies comparing the prevalence of SARS-CoV-2 infection and Covid-19 mortality with whole country serum 25(OH)D levels

From April 2020, the first SARS-CoV-2-specific data supporting a role for low vitamin D levels in SARS-CoV-2 infection and Covid-19 disease severity began to appear in the peer-reviewed literature. Initially, these were observational ecological studies which noted an increased incidence of infection and death from Covid-19 in countries where vitamin D deficiency (or low sun exposure) was common. For example, one study observed a 4.4% increase in risk of mortality from Covid-19 for every 1 latitudinal degree north of 28 degrees N (*p* = 0.031) after adjustment for population age profile [24], while another observed a statistical tendency towards correlation between mean population 25(OH)D levels and SARS-CoV-2 incidence/million population (*rho* 0.44; *p* = 0.05) and Covid-19 mortality/million population (*rho* 0.44; *p* = 0.05) across 20 European countries [25]. These data suggested that, at least at a population level, the spread of SARS-CoV-2 infection and the severity of Covid-19 disease within a population were linked to population vitamin D status.

Further supportive evidence of a link between vitamin D status and Covid-19 illness began to emerge in May 2020. This literature can be broadly categorised into studies examining vitamin D status and risk of SARS-CoV-2 infection, and studies examining vitamin D status and severity of Covid-19 disease.

### Studies comparing the rate of SARS-CoV-2 detection according to vitamin D status

In a Swiss report of 107 adult hospital admissions (mean age 73 years), 25(OH)D levels were significantly lower in SARS-CoV-2 PCR-positive patients (median 27.8 nmol/l) than in SARS-CoV-2 PCR-negative patients (median 61.5 nmol/l) (*p* = 0.004) [26]. In a study of 7807 Israeli adults, 25(OH)D levels were also lower amongst SARS-CoV-2-positive patients than SARS-CoV-2-negative patients. Adjusted odds ratios of 1.45 and 1.95 were reported for SARS-Cov-2 infection and Covid-19 hospitalisation, respectively, in patients with 25(OH)D levels < 75 nmol/l vs. > 75 nmol/l after adjustment for age, demographics, and underlying disease. The authors concluded that lower serum vitamin D levels appeared to be an independent risk factor for SARS-CoV-2 infection and Covid-19 hospitalisation [27].

Further observational studies concur with these findings. A large Israeli study comparing 52,405 SARS-CoV-2-positive cases and 524,050 controls reported lower 25(OH)D levels in the SARS-CoV-2-positive cohort, even after adjustment for ethnicity, geographic location, and gender (*p* < 0.001). This study also highlighted a 27% increased risk of SARS-CoV-2 infection with 25(OH)D < 30 nmol/l vs. 25(OH)D > 75 nmol/l after adjustment for ethnicity (*p* < 0.001), and suggested that recent supplementation was associated with lower infective risk [28].

Further recent data from the USA underscore the association between serum vitamin D levels and SARS-CoV-2 positivity. In a Chicago study of 489 patients with suspected SARS-CoV-2 infection, 71 tested positive by nasal swab PCR. After adjustment for age and non-white ethnicity, those with confirmed or likely low vitamin D status over the previous year had a 77% higher risk of testing positive for SARS-CoV-2 than their vitamin D-replete peers [29]. Subsequent US data from 191,000 patients from across all 50 states for whom vitamin D levels were available within the previous 12 months showed that those with serum 25(OH)D < 50 nmol/l had a 54% higher risk of PCR-confirmed SARS-CoV-2 positivity than those with levels of 75–85 nmol/l, and a 112% higher risk of PCR-confirmed SARS-CoV-2 positivity than those with levels > 137 nmol/l, with a calculated adjusted odds ratio for infection of 0.984 per 2.5 nmol/l increment in 25(OH)D concentration which persisted on multivariable analysis. The authors concluded: “SARS-CoV-2 positivity is strongly and inversely associated with circulating 25(OH)D levels, a relationship that persists across latitudes, races/ethnicities, both sexes, and age ranges.” [30].

### Case-control studies examining clinical outcome in Covid-19 patients according to vitamin D status

A number of case-control studies have examined the association between vitamin D deficiency and severity of Covid-19 disease. For example, one early US study found that 84.6% of ICU Covid-19 patients vs. 57.1% of Covid-19 ward patients (and typically ~ 40% of general ICU patients) had serum 25(OH)D < 75 nmol/l. Moreover, 100% of Covid-19 patients under 75 years who were admitted to the ICU in this study had serum 25(OH)D < 75 nmol/l. Overall, mean 25(OH)D was 48 nmol/l amongst the Covid-19 patients admitted to ICU vs. 74.5 nmol/l in the Covid-19 patients who did not require ICU admission [31].

Amongst older adults specifically, a further UK study examined severity of Covid-19 infection in 105 patients aged 65 years or over presenting with Covid-19 signs and symptoms. Patients who were confirmed SARS-CoV-2-positive by nasal swab viral reverse transcriptase PCR or who had characteristic radiological evidence of Covid-19 disease (*n* = 70) had a median serum 25(OH)D of 27 nmol/l compared with a median 25(OH)D of 52 nmol/l in the remaining 35 patients who did not have confirmed infection (*p* = 0.0008). Covid-19 patients with serum 25(OH)D < 30 nmol/l were also significantly more likely to require ventilation and ICU admission than those with 25(OH)D > 30 nmol/l (30.8% vs. 9.7%; OR 4.15, *p* = 0.042) [32].

Here in Ireland, an early pilot study in Dublin examined vitamin D status and clinical course in 33 male Covid-19 patients aged 40 years and over. None of these patients had cancer, diabetes mellitus, cardiovascular disease, or had received chronic immunosuppressive therapy. Amongst the 12 patients admitted to ICU (mean age 60 years), mean serum 25(OH)D was 27 nmol/l, while amongst the 21 patients who did not require ICU admission (mean age 56 years), mean 25(OH)D was 41 nmol/l (*p* = 0.03). These levels compared with a mean 25(OH)D of 47 nmol/l for males aged 40–60 years in Dublin and support the idea that these Covid-19 patients had lower vitamin D levels than males of a similar age during the same season, and that vitamin D levels were particularly low amongst the most severely affected subgroup of patients who required ICU admission. In terms of risk, this study calculated a 3.2 times greater likelihood of intubation amongst those with serum 25(OH)D < 30 nmol/l [33].

More recent data from Germany echo these findings. In a cohort of 185 SARS-CoV-2-positive patients, mean serum 25(OH)D level was significantly lower in the 93 subjects with Covid-19 illness who required hospital admission than in the remaining 92 SARS-CoV-2-positive subjects who did not require hospitalisation (36.5 nmol/l vs. 46.5 nmol/l) (*p* = 0.001), with 31% of inpatients vs. 13% of outpatients recording 25(OH)D < 30 nmol/l (*p* = 0.004). Critically, amongst the 93 admitted patients, even after adjustment for age, gender, and underlying disease (diabetes, cardiovascular disease, kidney disease, lung disease, and cancer), the risk of invasive mechanical ventilation was 6.1 times higher and the risk of death 14.7 times higher in those with 25(OH)D < 30 nmol/l vs. those with 25(OH)D greater than this 30 nmol/l threshold (*p* < 0.001 for both). Additionally, the significantly higher IL-6 levels observed in the vitamin D-deficient group suggested that their low vitamin D levels might have amplified the inflammatory response to infection which causes organ dysfunction [34].

### Intervention studies showing reduced disease severity and reduced mortality in Covid-19 patients receiving vitamin D supplementation

While the observational data presented above are persuasive, and reaffirm previous associations between low vitamin D status and the characteristics which portend poorer outcome in SARS-CoV-2 infection, they do not, in and of themselves, demonstrate causality. As we await the outcome of large prospective RCTs which are ongoing in the USA, France, Italy, and Spain, however, two small prospective intervention trials have recently reported.

A pilot intervention study from Cordoba, Spain, examined the clinical course of 76 consecutive patients (mean age 53 years) hospitalised with Covid-19 illness. All patients received hydroxychloroquine and azithromycin over the first 5 days of their admission and were randomised in a 2:1 ratio, such that 50 patients also received 532 μg of calcifediol (25(OH)D) on day 1, and 266 μg of calcifediol on day 3 and day 7, while the remaining 26 patients did not. Over the course of their admission, 1 patient from the calcifediol group (i.e. 2%) was admitted to ICU vs. 13 patients (i.e. 50%) from the unsupplemented group in which two patients died. This equates to an odds ratio of 0.03 for ICU admission in the calcifediol group, even after adjustment for pre-existing diabetes and high blood pressure. In simple terms, these pilot data suggest a 25–30-fold reduced risk of ICU admission amongst patients who received the calcifediol intervention [35].

Apart from these morbidity data, mortality data have emerged very recently showing a clear signal between bolus vitamin D supplementation and enhanced survival amongst French nursing home residents with Covid-19 illness. Notwithstanding the small size of this cohort (*n* = 57 in the vitamin D intervention group and *n* = 9 controls), vitamin D supplementation was strongly associated with enhanced clinical improvement scores for Covid-19 (*p* = 0.001). More pertinently, on follow-up at 5 weeks, 82.5% of the supplemented group had survived vs. just 44.4% of the unsupplemented controls (*p* = 0.023), with a fully adjusted mortality hazard ratio of 0.11 in those supplemented with vitamin D [36].

## Putative protective mechanisms

We now have a greater understanding of the putative protective effects of vitamin D against SARS-CoV-2 infection and Covid-19 illness; evidence which further underscores the plausibility of vitamin D deficiency as a causal effector of increased SARS-CoV-2 infection and Covid-19 disease severity.

### Infection

In relation to infection, the regulatory effects of vitamin D in optimising innate and adaptive immune function have been comprehensively reviewed by Greiller and Martineau (2015) and others [12, 37, 38]. These include enhanced production of cathelicidins (antimicrobial peptides associated with viral and bacterial surface disruption); amplified β-defensin expression (genes responsible for the production of antimicrobial peptides); enhanced macrophage phagocytosis and efferocytosis (efficient removal of cellular debris preventing further inflammation); and regulation of the macrophage oxidative burst (eliciting a more potent and more quickly resolved oxidative surge). These effects improve the efficiency with which respiratory pathogens including viruses are eradicated.

Furthermore, while existing data had already shown that vitamin D enhances the anti-viral activity of bronchial epithelial cells in vitro [39], recent research examining 2191 candidate compounds also identified a potential role for vitamin D in the specific inhibition of Covid-19 viral replication. The authors of this study concluded that calcitriol exhibits “significant, potent anti-viral activity” against SARS-CoV-2 in cultured human nasal epithelial cells in vitro [40].

### The restorative effects of vitamin D may enhance recovery during infection

Regarding disease severity, recent literature has described the modulatory effects of vitamin D in disease-related inflammation, including that related to viral respiratory infection. Emerging evidence suggests that these effects are critical in attenuating the cytokine storm which precipitates poorer outcome in Covid-19 patients. For example, vitamin D’s suppression of the renin-angiotensin system (RAS), and specifically its restoration of ACE2, is thought to reduce levels of pro-inflammatory angiotensin II while simultaneously increasing levels of anti-inflammatory angiotensin I–VII [41, 42]. There is also evidence that vitamin D, partly through its effects on the RAS, may mediate further anti-inflammatory, anti-thrombotic effects via the *kinin-kallikrein* system [43]. Vitamin D also has direct suppressive effects on several inflammatory cytokines centrally implicated in fulminant Covid-19 illness, including interleukin-6 (IL-6) [44], tumour necrosis factor-alpha (TNF-α) [13], and interferon-gamma (IFNγ) [14].

The avidity of vitamin D metabolites for vitamin D binding protein (VDB) is also pertinent to the inflammation which characterises severe Covid-19 disease. VDB is a pleiotropic protein which apart from its role in vitamin D metabolism also sequesters intracellular globular cytoskeletal proteins including actin, when they are liberated during tissue injury. The resulting VDB/actin complexes potentiate neutrophil chemotaxis and macrophage activation at the site of injury causing further inflammatory damage [45, 46]. By optimising vitamin D status and sequestering VDB, circulating free-VDB levels are reduced, ultimately limiting the generation of these pro-inflammatory VDB/actin complexes.

The production of type I interferons (IFNs) is an essential element of the innate immune response which restricts viral replication and spread. Reports have suggested that SARS-CoV-2 infection is associated with an absent or delayed type I IFN response and an exacerbated release of pro-inflammatory cytokines [47]. It is known that the SARS-CoV-2 virus encodes at least 10 proteins that allow the virus to inhibit, evade, or counteract the effects of type I IFNs [48]. In a small study by Trouillet-Assant et al., Covid-19 patients who failed to produce a detectable type I IFN response were significantly more likely to require invasive ventilation [49]. It has been shown that vitamin D potentiates the activity of type I interferons in hepatitis C infection [50] as well as during treatment for multiple sclerosis [51, 52]. These studies support the notion that vitamin D may augment early type I interferon responses in SARS-CoV-2 infection.

Finally, there is evidence that apart from these immunological effects, high-dose vitamin D supplementation in critical illness can elicit improvements in metabolomic profile. These favourable changes include increases in sphingomyelins, plasmalogens, lysoplasmalogens, and lysophospholipids, and decreases in acylcarnitine, phosphatidylethanolamine, and amino acid class metabolites; alterations which have collectively been associated with reduced 28-day mortality amongst ICU patients [53].

**Fig. 2:**
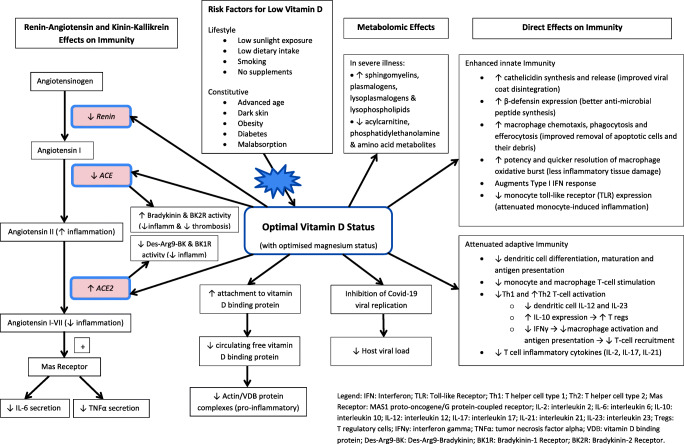
Role of vitamin D in the immunological, biochemical and metabolomic response to SARS-CoV-2 infection and illness

The putative immunological, biochemical and metabolomic mechanisms by which vitamin D elicits its protective effects against SARS-CoV-2 infection and Covid-19 disease severity are illustrated in Fig. 2.

### Summary of evidence

In summary, there are now multiple evidential strands linking low vitamin D status to increased SARS-CoV-2 infection and Covid-19 disease severity. These data include findings from ecological studies highlighting the high incidence of severe Covid-19 infection and death amongst groups and populations known to be at high risk of vitamin D deficiency, as well as epidemiological studies indicating lower risk of respiratory infection in general, and latterly, of SARS-CoV-2 infection specifically, in those with higher vitamin D levels. They also derive from prior intervention studies suggesting a reduced risk of some acute respiratory infections in patients who have taken vitamin D supplements, and from multiple studies showing an attenuation of inflammation, improved clinical outcomes, and reduced mortality in Covid-19 patients who are vitamin D replete. The recent emergence of pilot RCT data demonstrating reductions in ICU admission and mortality amongst hospitalised Covid-19 patients receiving vitamin D supplementation are particularly compelling. Finally, there is a wealth of literature describing the putative mechanisms by which vitamin D likely mediates these positive effects on both SARS-CoV-2 infection risk and Covid-19 disease severity. The increasing volume and concordance of these findings from diverse studies with varying methodologies in a multiplicity of settings and populations highlight vitamin D correction as a critical priority in the protection of vulnerable groups from the worst effects of Covid-19.

## Intervention

The data described herein present a compelling case for the optimisation of vitamin D status across the population, but particularly in vulnerable groups (e.g. those with obesity, those with darker skin, older adults, and those in institutional settings) where vitamin D deficiency is endemic. Implicit in this is the need for vitamin D supplementation, the safety profile of which is now well-established [54–56].

### Treatment of vitamin D deficiency with vitamin D supplementation: current vitamin D status in Ireland and target populations

#### Vitamin D intake in Irish adults

In Ireland, while national vitamin D intakes for older adults have not been reported in recent years, historic data from 1999 refer to median intakes of 2.9 μg per day amongst adults aged 18–64 years [57], and in a separate cohort, to mean daily intakes of 3.2–5.8 μg per day in post-menopausal Irish women [58]. More recent 2009 data for Irish adults aged 18–64 years have estimated a median daily intake of 3.5 μg per day, with just 16% of the population consuming vitamin D-containing supplements [59], further articulating the persisting deficits in vitamin D intake which prevail amongst the general population.

#### Vitamin D status in Irish adults

While the UK Scientific Advisory Committee on Nutrition (SACN) defines vitamin D deficiency as serum 25(OH)D < 25/30 nmol/l, most advisory bodies and expert groups, including the European Food Safety Authority, the Endocrine Society in the US and the European Calcified Tissue Society define deficiency at the < 50 nmol/l threshold [60]. This 50 nmol/l target appears to be consistent with the very minimum serum level required to enhance (but not necessarily optimise) immunity to viral respiratory infection including SARS-CoV-2 [20, 28, 30].

At this 50 nmol/l threshold, there are significant data describing a high prevalence of vitamin D deficiency across the Irish adult population, particularly in winter. For example, the last National Adult Nutrition Survey (NANS) revealed that 35.7% of adults aged 50–64 years, and 44.0% of adults aged 65–84 years, had 25(OH)D < 50 nmol/l, with these figures rising to ~ 55% and 48% respectively in winter [61]. More recent data describing vitamin D status in Dublin and surrounding counties show a high prevalence of deficiency across all age groups, with the highest rates recorded in the 18–39-year age category where 21% had 25(OH) < 30 nmol/l and 47% had 25(OH)D < 50 nmol/l over the 5-year period 2014–2018 [62].

Amongst older adults specifically, further data from the Irish Longitudinal Study on Ageing (TILDA) concur with the findings from NANS, identifying serum 25(OH)D < 50 nmol/l in 43% of community-dwelling adults aged 50 years and over, with the highest risk of deficiency observed in obese and physically inactive individuals, those of low SES, those with underlying conditions such as hypertension and diabetes mellitus, and those who did not take supplements [63]. There is also evidence that the prevalence of vitamin D deficiency increases with age amongst older Irish adults, with serum 25(OH)D < 30 nmol/l recorded in 22–37% of community-dwelling adults aged over 70 years [64], rising to 42% with serum 25(OH)D < 25 nmol/l amongst Irish nursing home residents [65].

Apart from age, obesity is a potent predictor of vitamin D deficiency. While Irish data describing differences in serum vitamin D according to anthropometric status are lacking, it is noteworthy that 60% of Irish adults are now overweight or obese, with these figures rising to 75% in the 65–74-year age group and 72% in the 75+ year age group in the most recent national survey [66]. It is also important to note that apart from its direct effects on vitamin D status, obesity is a principle determinant of many of the chronic diseases which have been explicitly linked to poorer outcome in Covid-19-related illness amongst older adults [67].

Collectively, these data suggest that while low 25(OH)D levels are observed in a very significant proportion of older adults, vitamin D deficiency (including severe deficiency) and the factors which precipitate it are ubiquitous across all age groups in Ireland, highlighting the need for a whole population response to this issue.

### Requirements

Ireland’s low vitamin D intakes from food significantly increase our reliance on cutaneous synthesis to achieve and maintain sufficient serum 25(OH)D levels. However, Ireland’s northerly latitude and consequent low or absent UVB irradiation at the required 290–315 nm militate against the achievement of adequate vitamin D status by sunlight exposure, particularly in wintertime. These deficits in dietary intake and cutaneous synthesis are the key drivers of Ireland’s widespread vitamin D deficiency, and call for increased vitamin D supplementation in Irish adults to ensure that serum 25(OH)D is corrected in a timely manner during the current pandemic.

Previous research has shown that in Ireland, total vitamin D_3_ intake of ~ 25–30 μg/day (1000–1200 IU/day) is required to reliably maintain 25(OH)D > 50 nmol/l on a year-round basis in adults aged 20–40 years [68], and in those aged 64 years and over [69]. However, it has been more recently argued in the USA that intakes of 37.5–50 μg/day (1500–2000 IU/day) are required for specific protection against SARS-CoV-2 infection and Covid-19 illness [30, 70]. Given our high national prevalence of obesity, it is possible that these higher requirements for protection against Covid-19 may also pertain amongst some vulnerable population groups in Ireland, as the dosage needed to achieve the same increment in serum 25(OH)D concentration is thought to rise by ~ 40% in obesity [71]. Indeed, one meta-analysis in this area has proposed that while 20 μg/day (800 IU/day) appears adequate to meet the 25(OH)D sufficiency threshold of 50 nmol/l in obesity, doses to achieve a serum target of 75 nmol/l in these individuals with obesity “would be much higher” (e.g. 84 μg/day (3360 IU/day) in a 100-kg subject with baseline 25(OH)D < 25 nmol/l) [72].

In Ireland, therefore, physiological vitamin D requirements for optimal immune function are considerably higher than those that can be achieved from food alone, further underpinning the need for supplementation.

### Safety

Under clinical supervision and in the absence of rare CYP24A1 enzyme deficiency [73, 74] or underlying granulomatous or other clinical conditions which increase 1α-hydroxylase activity (e.g. sarcoidosis, tuberculosis, some lymphoid tumours, certain kidney diseases) [75–77], prescription of oral vitamin D_3_ at doses of 20–25 μg/day (800–1000 IU/day) is safe and effective in achieving a minimum target serum 25(OH)D of at least 50 nmol/l in most adults. This is particularly important in older adults and other vulnerable population groups in order to address the winter nadir in vitamin D status [78]. These doses are a quarter or less of the 100 μg/day (4000 IU/day) tolerable upper limit for daily intake cited by the Institute of Medicine (IoM) in the USA [79] and the European Food Safety Authority (EFSA) [80] (i.e. less than a quarter of “the maximum level of total chronic daily intake of a nutrient (from all sources) judged to be unlikely to pose a risk of adverse health effects to humans” [81]). They are predicated on an average incremental rise of 0.6–0.7 nmol/l in serum 25(OH)D for each additional μg/day (40 IU/day) of oral vitamin D_3_ articulated by previous kinetic studies in this area [82]. In individuals with obesity, those with darker skin and in older adults, especially those in nursing homes and other institutional settings, it is likely that doses higher than this will be required to reach the required 25(OH)D level for optimal immune function [83]. These higher doses should be titrated according to baseline 25(OH)D levels and are safe under medical supervision on the premise that the dose response curve flattens significantly above a daily oral dose of 35 μg/day (1400 IU/day) [84].

The doses which we are recommending fall well within the oral doses of vitamin D shown to be safe in other studies [54, 56], including recent large-scale intervention trials which supplemented with 50 μg/day (2000 IU/day) [85] and 100 μg/day (4000 IU/day) [86] of vitamin D_3_ over extended durations of 2 to 5 years and showed no increased risk of hypercalcaemia, renal stones, or other adverse outcomes. They are also significantly less than the equivalent vitamin D “dose” generated by cutaneous synthesis in the presence of UVB irradiation from summer sunlight [87]. Furthermore, in the recent publication by Scully et al. [62] which examined vitamin D levels in 36,466 participants (age 18–109 years) from 28 geographical areas across Dublin, Wicklow, and Kildare, there were only 21 patients with vitamin D levels ≥ than 250 nmol/l. The highest corrected calcium level amongst these 21 patients was 2.47 nmol/l — none had hypercalcaemia.

## Conclusion

The current pandemic has claimed the lives of over 1800 people in Ireland, half of them vulnerable older adults, and continues to pressurise our acute care services. While it would be preferable to have large-scale randomised control trial data to conclusively prove the case for vitamin D supplementation as a protectant against SARS-CoV-2 infection and Covid-19 illness, this is extremely challenging in a quickly evolving pandemic. Given the strong emerging evidence which suggests a protective role for vitamin D against Covid-19, the proposition of future randomised control trials incorporating a non-intervention arm which includes those with baseline vitamin D deficiency in whom there is an existing clinical imperative to intervene is untenable from an ethical perspective. Conducting such randomised placebo-controlled trials only in those who are vitamin D replete is futile as it cannot address the question of clinical efficacy in those who have low vitamin D levels, and who would be the target of any proposed intervention. So while data from well-designed, prospective randomised control trials would provide definitive evidence in this area, these are, and may remain, elusive. We are consequently reliant on data which are imperfect in isolation, but which in their totality, present compelling evidence for a protective effect of vitamin D against Covid-19, and which strongly support urgent intervention in this regard.

In this context, reflecting on lessons learned in managing the Ebola outbreak in Africa, Dr. Michael Ryan, Executive Director of WHO, captured the imperative to act decisively in the current Covid-19 crisis: “Be fast, have no regrets. You must be the first mover. The virus will always get you if you don’t move quickly; if you need to be right before you move, you will never win”. He went on to conclude, “Perfection is the enemy of the good when it comes to emergency management; speed trumps perfection. The problem in society we have at the moment is that everyone is afraid of making a mistake. Everyone is afraid of the consequence of error. But the greatest error is not to move. The greatest error is to be paralysed by the fear of failure.” [88].

## Recommendations

A majority of the Irish adult population have insufficient vitamin D intake and a very significant proportion across all age groups have low serum vitamin D levels. This should be addressed as a matter of urgency. Without vitamin D supplementation, the blood levels associated with protection against severe viral respiratory infection due to SARS-CoV-2 will not be achieved. While food fortified with vitamin D is recommended as a first-line strategy to augment intakes, for the vast majority, vitamin D supplements will also be required. The evidence linking vitamin D deficiency with increased risk of SARS-CoV-2 infection and Covid-19 disease severity has evolved significantly since March 2020, and now strongly supports the need for intervention in this area. Given this evidence and the unambiguous safety profile of daily intakes at these levels, we recommend that adults in Ireland should be supplemented with oral vitamin D_3_ at 20–25 μg/day (800–1000 IU/day) for the duration of this pandemic. For those who are overweight or obese, or who have dark skin pigmentation or other risk factors for vitamin D deficiency, it is likely that supplementation at daily doses higher than this will be required to achieve the serum 25(OH)D levels needed for optimal immunity against Covid-19. In these groups and in older adults, amongst whom vitamin D deficiency and severe deficiency (i.e. 25(OH)D < 25/30 nmol/l) prevail, and for whom SARS-CoV-2 infection carries significantly greater clinical risk, prescription at these higher daily doses according to baseline serum 25(OH)D should proceed as required under medical supervision. This is especially important for older adults resident in nursing homes or other long-term care settings who are particularly vulnerable; here, sufficient vitamin D supplementation to achieve a minimum serum 25(OH)D level of 50 nmol/l should be expeditiously implemented as a priority element of standard care.
